# Correlative cryo super-resolution light and electron microscopy on mammalian cells using fluorescent proteins

**DOI:** 10.1038/s41598-018-37728-8

**Published:** 2019-02-04

**Authors:** Maarten W. Tuijtel, Abraham J. Koster, Stefan Jakobs, Frank G. A. Faas, Thomas H. Sharp

**Affiliations:** 1 Section Electron Microscopy, Dept. of Cell and Chemical Biology, Leiden University Medical Center, 2300 RC Leiden, The Netherlands; 2 NeCEN, Gorlaeus Laboratories, Leiden University, 2333 CC Leiden, The Netherlands; 3 Max Planck Institute for Biophysical Chemistry, Dept. of NanoBiophotonics and University Medical Center of Göttingen, Dept. of Neurology, Am Faßberg 11, 37077 Göttingen, Germany

**Keywords:** Cryoelectron microscopy, Super-resolution microscopy

## Abstract

Sample fixation by vitrification is critical for the optimal structural preservation of biomolecules and subsequent high-resolution imaging by cryo-correlative light and electron microscopy (cryoCLEM). There is a large resolution gap between cryo fluorescence microscopy (cryoFLM), ~400-nm, and the sub-nanometre resolution achievable with cryo-electron microscopy (cryoEM), which hinders interpretation of cryoCLEM data. Here, we present a general approach to increase the resolution of cryoFLM using cryo-super-resolution (cryoSR) microscopy that is compatible with successive cryoEM investigation in the same region. We determined imaging parameters to avoid devitrification of the cryosamples without the necessity for cryoprotectants. Next, we examined the applicability of various fluorescent proteins (FPs) for single-molecule localisation cryoSR microscopy and found that all investigated FPs display reversible photoswitchable behaviour, and demonstrated cryoSR on lipid nanotubes labelled with rsEGFP2 and rsFastLime. Finally, we performed SR-cryoCLEM on mammalian cells expressing microtubule-associated protein-2 fused to rsEGFP2 and performed 3D cryo-electron tomography on the localised areas. The method we describe exclusively uses commercially available equipment to achieve a localisation precision of 30-nm. Furthermore, all investigated FPs displayed behaviour compatible with cryoSR microscopy, making this technique broadly available without requiring specialised equipment and will improve the applicability of this emerging technique for cellular and structural biology.

## Introduction

Cryo electron microscopy (cryoEM) provides *in-situ* structural information of biological samples at high resolution, but it is essential that samples are fixed in their near-native state by vitrification. Although cryoEM is a powerful technique, it is challenging to locate specific proteins and regions of interests at low magnification. Cryo-fluorescence light microscopy (cryoFLM) is compatible with cryoEM and can provide protein localisation of individually labelled biomolecules within a large field of view^1–5^. To maintain the native state of the sample the use of external labelling steps, membrane permeabilization or the addition of cryoprotectants is precluded. Labelling steps can be avoided by utilising genetically encoded fluorescent proteins (FPs)^6^. An emerging workflow for cryoCLEM consists of locating areas of interest using cryoFLM, followed by inspecting the sample at high resolution using cryoEM^1,2^. However, the resolution gap between the two modalities makes it challenging to directly correlate a cryoFLM signal to a particular structure imaged with cryoEM. Specifically, to maintain sample integrity, cryoFLM currently utilizes long working-distance objective lenses, with a concomitant lower numerical aperture, limiting the lateral resolution to ca. 400 nm^2,7,8^, although the resolution may be improved by to the recent development of immersion fluids suitable for cryoFLM^9^.

Super-resolution (SR) microscopy techniques^8,10,11^, such as single-molecule localisation microscopy (SMLM)^12,13^ have been used for over a decade to increase the resolution of diffraction-limited room temperature (RT)-FLM by an order of magnitude, and localisation precisions in the nanometre-range can be achieved^14,15^. With the increased resolution of RT-SR microscopy comes a similar sensitivity to sample preparation artefacts or other alterations from the native state of the sample. For example, chemical fixation is known to disrupt cellular structures, such as microtubules and mitochondria^16^. Furthermore, chemical fixation does not always immobilise the entirety of the sample and, for instance, membrane proteins are known to retain some mobility even after chemical fixation^17,18^. Cryofixation provides superior sample fixation, which is less prone to artefacts, so adapting SR to vitrified cryosamples poses a valuable alternative to RT-SR^8^. Furthermore, the reduced photobleaching rate of fluorophores at low temperatures^5,19–21^ can increase the photon yield of single-molecules, leading to a sub-nanometre localisation precision^19,22^.

Several workflows have previously been developed to combine SR microscopy and EM^23–26^. Combining these techniques is challenging, as the fixation and staining protocols for EM are often not compatible with fluorescence^21,27^. Furthermore, these methods suffer from the use of heavy-metal staining instead of directly imaging biomaterial, as is done with cryoEM. Here, we have developed methodology to increase the resolution of cryoFLM by addressing the challenges that arise when applying SMLM to cryosamples. Firstly, instabilities caused by the increased mechanical and thermal drift of cryostages must be mitigated^7^. Previously, this has been achieved by physically adapting cryostages to reduce drift in addition to image registration after acquisition^28,29^. Alternatively, drift can be actively countered during acquisition by using a bespoke system that tracks the movement of fiducial beads and counters this movement by driving a piezo sample stage^30,31^. Secondly, SMLM requires high excitation intensities on the sample to maximise the number of emitted photons, which directly influences the localisation precision^32^. However, it has been shown that intense illumination can cause sample-devitrification and damage^28,30^, and precludes subsequent imaging using cryoEM. Carbon film, which is commonly used as a support material for cryoEM, absorbs the incoming light and converts the radiation to heat, thereby raising the temperature of the vitrified sample above the devitrification temperature (ca. 133 K), causing devitrification. Devitrification can be mitigated by lowering the intensity of the excitation laser or shortening the duration of illumination by employing pulsed-illumination schemes^28,30^ or by the addition of cryoprotectants to the sample^28,30^. Although cryo protectants can be effective in preventing devitrification, it is not always possible to add them to the sample and they are known to lower the signal-to-noise ratio of cryoEM images^33^. In addition, pulsed illumination schemes increase acquisition time, which is not desirable as it increases the influence of drift and likelihood of sample contamination. Alternatively, the carbon support film can be replaced by more transparent materials, such as plastics like formvar^30^. However, these materials have a high fluorescent background and require ultraclean fabrication to be compatible with cryoFLM^30^.

Reversibly photoswitchable (RS) FPs have the ability to transfer between a dark inactive and a fluorescent active state^34–36^ and can be utilised to perform SMLM by independently imaging and localising single FPs multiple times^37,38^. Reversible photoswitching involves *cis*-*trans* isomerisation and protonation of the chromophore^39^, although a precise mechanistic understanding remains elusive, and may differ for each individual FP. Furthermore, there is currently no consensus whether RSFPs retain their photoswitching behaviour after vitrification and at low temperatures^28,30,40,41^. For example, various FPs (including Dronpa) cooled down in the presence of varying concentrations of cryoprotectants or in a non-vitreous environment displayed photoswitching, although less efficient compared to RT^30,40,42^. However, when FPs (including Dronpa) were investigated encapsulated inside vitrified bacteria at 80 K, only PA-GFP responded to photoactivation^28^. Furthermore, structural studies have shown that while the chromophore inside Padron can isomerise between the *cis* and *trans* state at 100 K while crystallised, for other FPs, such as Dronpa, mTFP0.7 and IrisFP, isomerisation when vitrified is prohibited by several amino acid residues blocking this reorganisation^42^.

We describe cryoSMLM acquisition using RSFPs and subsequent cryoEM imaging without the addition of any cryoprotectants and exclusively using commercially available equipment and sample supports. We found that cryosamples in our setup could be constantly illuminated for extended periods using a laser intensity of 28.5 W/cm^2^ without devitrification, although this greatly depends on sample and the support grid used. Furthermore, we systematically investigated several photophysical properties of various FPs under vitreous conditions at 77 K and found that all investigated FPs retain, or even gain, photoactivatability. These findings greatly improve the general applicability of our approach. We demonstrate the utility of this method by performing SR-cryoCLEM on RSFP-labelled lipid nanotubes and on intact mammalian cells to achieve a localisation precision of ca. 30 nm, with subsequent high-resolution imaging and correlation using cryoEM.

## Results

### Devitrification

**Figure 1:**
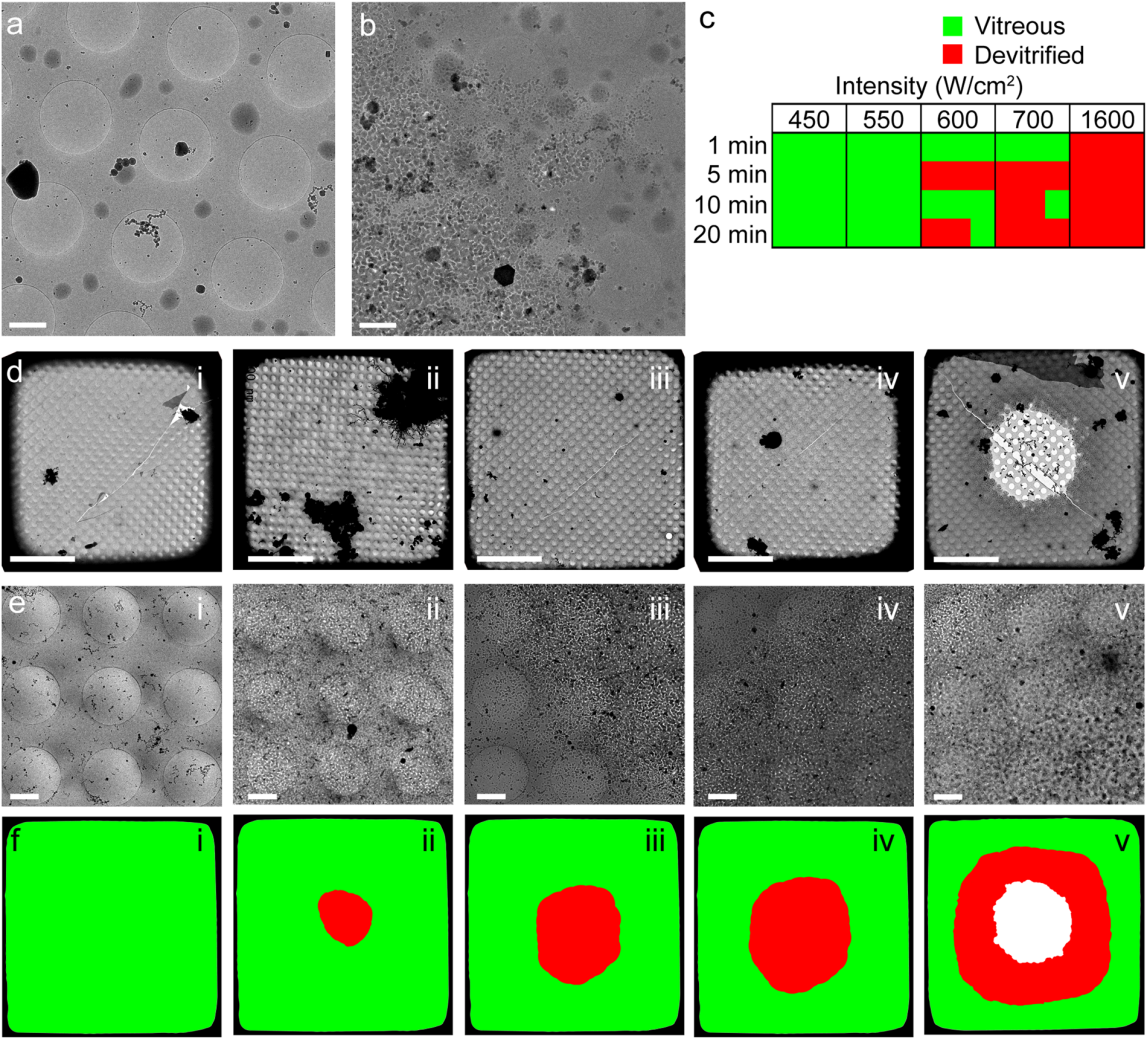
Damage of vitreous water caused by 488 nm laser illumination. The specimen shown is a thin holey-carbon film with regularly spaced circular openings supporting a thin film of vitrified water. (**a**) After 30 minutes of constant illumination using an intensity of 28.5 W/cm^2^, the ice remains vitreous. (**b**) When increasing the intensity to 33.7 W/cm^2^, after 5 min constant illumination a clear region of devitrified ice is observed. (**c**) Assessing laser-induced damage of vitreous water after constant illumination using various intensities and durations. For each condition, three replicates are shown. (**d**,**e**) Low (**d**) and high (**e**) magnification cryoEM images of vitreous water illuminated for 60 sec at the following intensities: i, 31.1 W/cm^2^; ii, 41.5 W/cm^2^; iii, 51.9 W/cm^2^; iv, 62.2 W/cm^2^; v, 83.0 W/cm^2^. (**f**) Schematic representation of vitreous (green), devitrified (red) or sublimated/dry (white) areas, corresponding with panels d and e. Scalebars: 1 µm in a,b,e and 20 µm in d.

Exposure to high intensity laser light can devitrify cryosamples^28,30^, which is clearly visible using low dose cryoEM (Fig. 1a,b). We sought to ascertain which continuous illumination intensity was suitable for imaging cryosamples without the addition of cryoprotectants. We found that devitrification is dominated by the laser intensity, and not the illumination time (Fig. 1c), and when illumination intensity remained at or below 28.5 W/cm^2^ ca. 95% of examined areas remained vitreous. This intensity is relatively low compared to intensities for RT-SMLM, which range from several hundred W/cm^2^ up to 100 kW/cm^2^ ^43,44^. Furthermore, devitrification invariably started as a region of crystalline ice in the centre of the grid squares, which increased in size with increasing illumination intensity (Fig. 1d–f). For the remainder of this work, illumination intensity did not exceed 550 W/cm^2^.

### Photophysical properties of FPs vitrified at 77 K

We tested 7 different FPs that were chosen for their diverse photoactivation properties at 294 K (Table 1). These were: EGFP^45^, which is very poorly photoactivatable^46^; the irreversibly-photoactivatable FP PA-GFP^46^; Dronpa^35^, rsFastLime^47^, rsEGFP2^15^ and mIrisFP^48^, which are all negatively reversibly photoactivatable with different switching properties; and Padron^14^, a positively RSFP (see Table 1 for further details). These (RS)FPs were assessed for their applicability when vitrified and evaluated at 77 K by measuring emission spectra, photoactivation and deactivation properties. Conflicting reports exist on the switchability of RSFPs at low temperatures^28,30,40^, perhaps due to the addition of cryo-protectants or limited laser intensity used to perform photoswitching. We adapted our cryoFLM system to accommodate a photospectrometer (see Materials and Methods), which allowed us to measure the emission spectra of vitrified FPs at 77 K, as well as at 294 K (Supplementary Fig. S1a). Previously, up to 60% of glycerol has been used to measure emission spectra^30,40^, but we found the addition of 50% glycerol resulted in a large (10.2 nm) shift of the emission spectrum maximum (Supplementary Fig. S1b). The emission spectra of vitrified samples at 77 K demonstrated blue-shifts of ca. 3.4 nm (SD = 2.6, n = 6) *cf*. 294 K. This small shift allowed us to use the same emission filters that are used based on the characteristics of FPs at 294 K.

**Table 1:** Fluorescent proteins used in this study.

Fluorescent Protein	Type	Reference
EGFP	Not photoactivatable	Cormack *et al*.^45^
PA-GFP	Irreversibly photoactivatable	Patterson *et al*.^46^
Dronpa	Negative Reversibly photoswitchable	Ando *et al*.^35^
Padron	Positive Reversibly photoswitchable	Andresen *et al*.^14^
rsFastLime	Negative Reversibly photoswitchable	Stiel *et al*.^47^
rsEGFP2	Negative Reversibly photoswitchable	Grotjohann *et al*.^15^
mIrisFP	Negative Reversibly photoswitchable and photoconvertible	Fuchs *et al*.^48^

At 294 K, EGFP is not photoactivatable. PA-GFP is singly photoactivatable, so molecules can be photoactivated once, and are then irreversibly photobleached. Dronpa, rsFastLime, rsEGFP2 and mIrisFP are positive reversibly photoswitchable FPs, so they can be reversibly photoactivated, and deactivated using light of the same wavelength as is used for excitation (i.e. the FPs are deactivated whilst being imaged). Conversely, Padron is a positive reversibly photoswitchable FP, which is photoactivated with the same wavelength as the excitation wavelength (i.e. Padron is activated whilst being imaged). Furthermore, mIrisFP is also photoconvertible, so upon illumination the emission can switch from green to red light.

**Figure 2:**
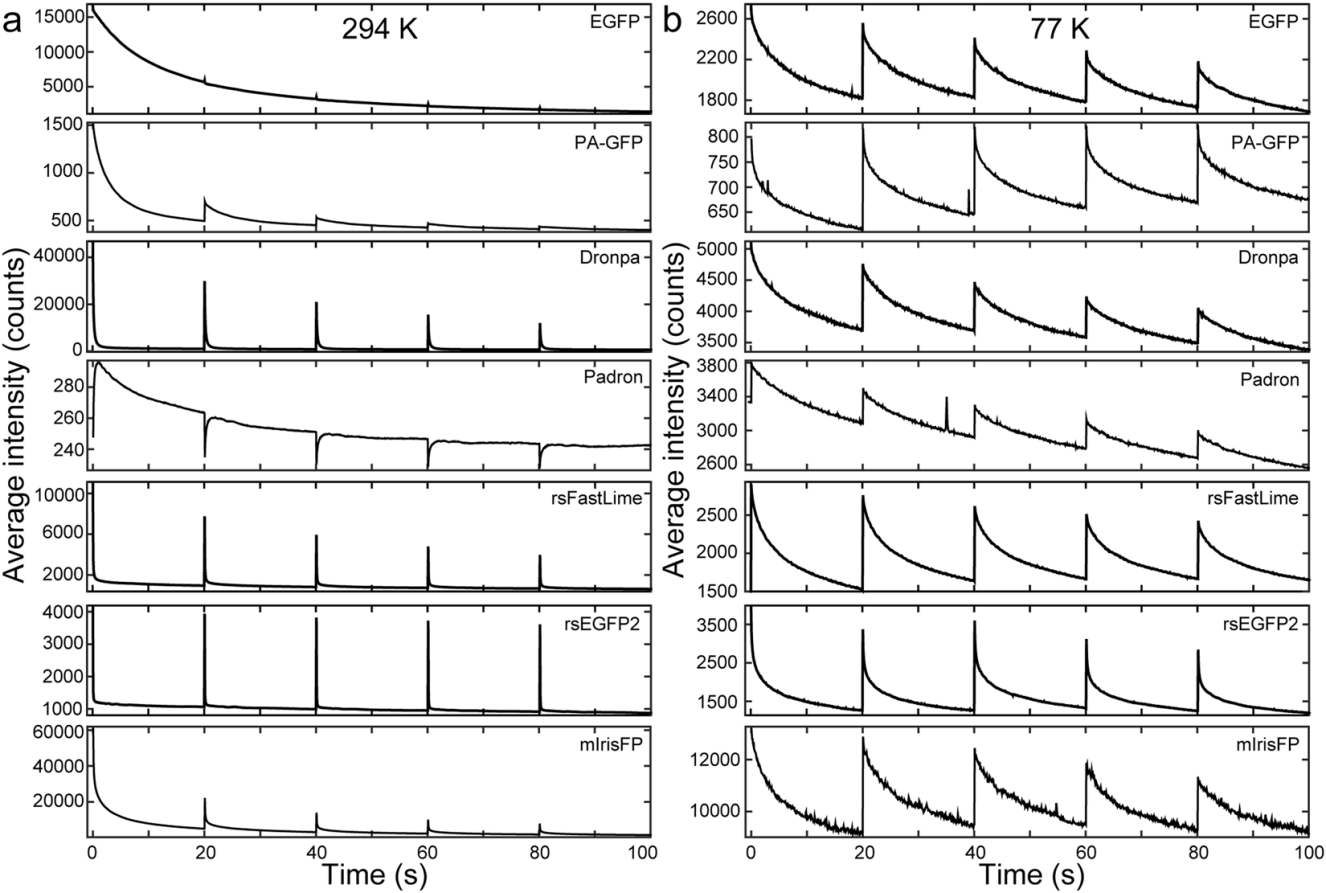
Photoactivation of fluorescent proteins at 294 K and 77 K. (**a**) Photoactivation of fluorescent proteins at 294 K. Fluorescent probes were activated with a 2.5 sec. 405 nm laser pulse every 20 sec, and fluorescence was monitored using excitation at 488 nm. (**b**) Identical photoactivation as shown in **a**, but at 77 K of vitrified fluorescent proteins on EM grids.

**Figure 3:**
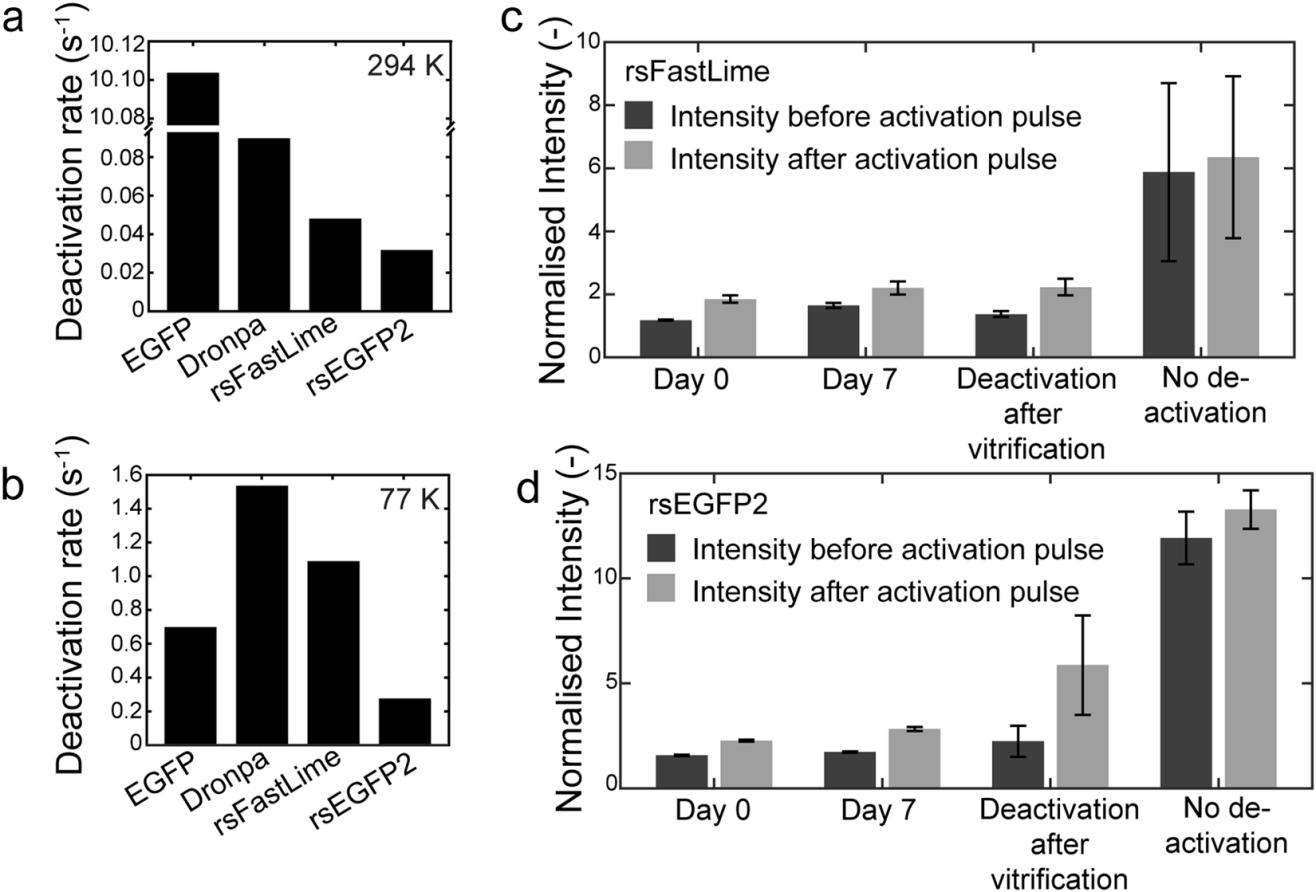
Deactivation of fluorescent proteins at 294 K and 77 K. The first deactivation curve of traces shown in Fig. 2 were fitted with a (bi-)exponential function, of which the fast rate is plotted in (**a**) and (**b**) for 294 K (aqueous) and 77 K (vitreous) respectively. (**c**) and (**d**) Deactivation prior to vitrification of FPs leads to retention of the deactivated state for rsFastLime and rsEGFP2, respectively. Following vitrification, fluorescent intensity was measured after 0 and 7 days, and the mean intensity before (dark grey) and after (light grey) a photoactivation pulse are shown. Deactivation for 20 min after vitrification of the FPs is shown, as well as photoactivation without any deactivation.

Next, we used our cryoFLM setup to measure photoactivation and deactivation of vitrified FPs at 77 K and in aqueous buffer at 294 K. We ensured that at 294 K all FPs behave as previously described (Fig. 2a)^14,15,35,45–48^. To our surprise, when vitrified and imaged at 77 K all FPs displayed similar activation and deactivation profiles and kinetics (Fig. 2b), and all RSFPs retained their photoactivation and deactivation ability, in contrast to previous reports^28^ but in agreement with others^30,40^. Strikingly, EGFP and PA-GFP also became reversibly activatable when vitrified (Fig. 2b), in agreement with earlier work using EGFP^30^. Furthermore, Padron changed from a positively to a negatively switching RSFP (Fig. 2b), indicating that the same physical mechanism for photoactivation may occur for all investigated FPs at 77 K. However, all activation traces had higher backgrounds, and the deactivation rate was much lower compared with 294 K (Fig. 2). We fitted (bi-)exponential functions (see Materials and Methods) to the first deactivation curve shown in Fig. 2a,b, and compared the fast exponents. All RSFPs could be deactivated at 77 K, but the deactivation rate was significantly decreased (Fig. 3a,b and Supplementary Fig. S2). Deactivation rates of the negatively RSFPs (Dronpa, rsFastLime and rsEGFP2) followed the same trend at 294 K and 77 K (Fig. 3a,b); Dronpa was the slowest and rsEGFP2 the fastest RSFP at both temperatures. These data informed our decision to use the two fastest RSFPs (rsFastLime and rsEGFP2) in further experiments.

### Deactivation of RSFPs prior to vitrification

The decreased deactivation rate at 77 K *cf*. 294 K resulted in a higher background when imaging. Prior to cryoSR acquisition, all FPs must be deactivated by illuminating with the deactivation laser so that newly activated FPs can be individually localised. However, due to the decreased deactivation rate this can take up to 20 minutes for each imaging position on the grid. To reduce this time, we attempted to deactivate whole grids at 294 K using a white-light lamp with a blue filter (470–490 nm; see Materials and Methods) to illuminate the entire grid for 1 min immediately prior to vitrification. We compared the fluorescent intensity of a position with the intensity of the same position immediately after an activation pulse and found that both RSFPs tested (rsFastLime and rsEGFP2) retained their deactive state after vitrification (Fig. 3c,d). Furthermore, the deactivated state was retained for at least 7 days whilst stored at 77 K in liquid nitrogen (Fig. 3c,d), thus reducing the acquisition time for 10 regions on each grid by more than 3 hours.

### Cryo super-resolution correlative light and electron microscopy on lipid nanotubes

**Figure 4:**
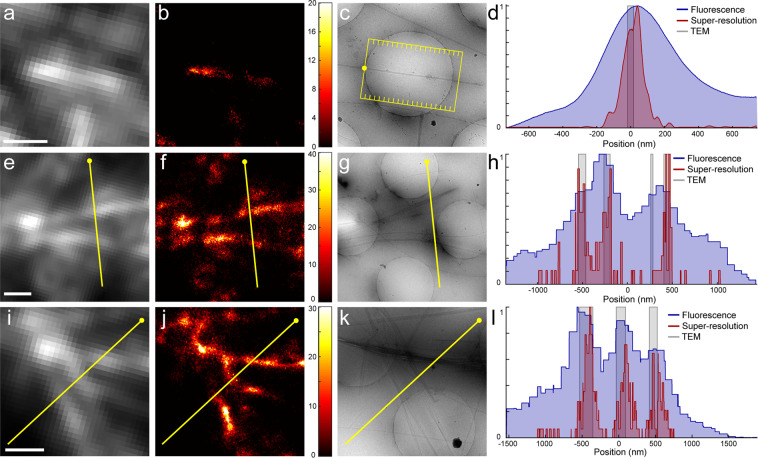
CryoCLEM using rsEGFP2-labelled lipid nanotubes. (**a**) Diffraction-limited cryoFLM image of a single lipid nanotube labelled with rsEGFP2. (**b**) SR image of the same region. The colourmap represents the number of localised single-molecules per SR pixel. (**c**) CryoEM image of the single tube overlaying a hole in the carbon support film. (**d**) Intensity profiles along the tube for each modality shown in (**a**–**c**). Multiple profiles were averaged perpendicular to the tube, indicated by the yellow box and lines in (**c**). (**e**) and (**i**) Diffraction-limited cryoFLM image of several bundled nanotubes labelled with rsEGFP2. (**f**) and (**j**), SR image of the same regions shown in **e** and **i**, respectively. (**g**) and (**k**), CryoEM image of the same regions SR image of the same regions shown in **e** and **i**, respectively. (**h**) and (**l**), Intensity profiles across the yellow line shown in (**e**–**g**) and (**i**–**k**). Profiles are drawn such that the yellow sphere corresponds to the left hand side in the profile plot. Scalebars: 1 µm.

We synthesised lipid nanotubes with 1 mol % Ni-NTA-conjugated lipids and labelled these with His-tagged rsFastLime or rsEGFP2 to generate samples suitable for SR-cryoCLEM. We performed cryoSMLM imaging as described in the Materials and Methods (see also Supplementary Fig. S3). Imaging lipid nanotubes labelled with rsEGFP2 showed photoactivation when embedded in vitreous ice (Fig. 4a–c). After image registration to counter instabilities during imaging, we could achieve a registration accuracy of ca. 10 nm (Supplementary Fig. S4 and Supplementary Note). Correlating this image with a cryoEM image of the same region showed an individual lipid nanotube spanning a hole in the carbon support film. Reconstruction yielded an average localisation precision of 30 nm for individual molecules (Supplementary Fig. 5 and Table S1). This increase in resolution was particularly notable when observing bundles of tubes overlaying the carbon support film (Fig. 4e–l and Supplementary Table S1); using cryoSR the distinction between three or two tube bundles becomes clear, while this could not be detected from the diffraction-limited cryoFLM image (Fig. 4e–h). Similarly, rsFastLime-labelled nanotubes showed photoactivation when embedded in vitreous ice (Supplementary Figs S6 and S7). Correlation with a cryoEM image of the same region showed a bundle of lipid nanotubes, and also revealed that the fluorescence was more intense over the holes of the carbon support film (Supplementary Fig. S7**)**. This is also apparent with rsEGFP2 (Fig. 4b,c), where the fluorescence intensity over the carbon support film is reduced compared to over the holes.

### Cryo super-resolution correlative light and electron microscopy on intact mammalian cells

**Figure 5:**
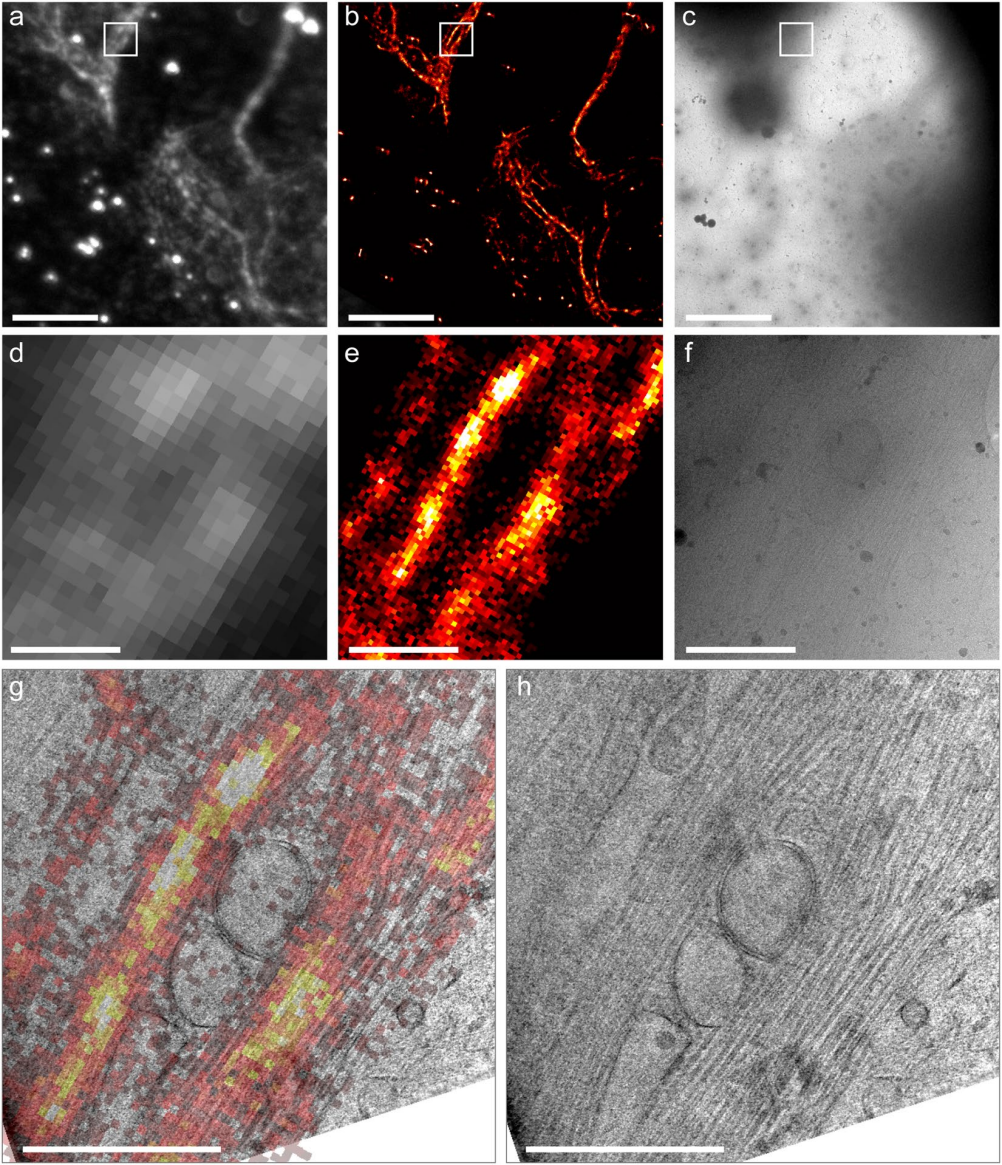
CryoCLEM on U2OS cells, transfected with rsEGFP2-MAP2. (**a**) Diffraction-limited cryoFLM image, (**b**) SR image and (**c**) CryoEM image of the same region, showing two positively transfected cells. (**d**–**f**) Higher magnification images of the region indicated with the white square in **a-c**, diffraction-limited cryoFLM, SR image and cryoEM image respectively. (**g**) Overlay of the SR image shown in **e** over an 18.6 nm thick slice through a tomographic volume of the sample in the same region. (**h**) An 18.6 nm thick slice through the tomographic volume of the sample in the same region as shown in (**f**), where the microtubule bundles can be clearly distinguished. Scalebars: 10 µm, in (**a**–**c**); 1 µm in (**d**–**h**).

To demonstrate SR-cryoCLEM on mammalian cells, we cultured human bone osteosarcoma epithelial (U2OS) cells on top of EM grids and transfected the cells with plasmid encoding rsEGFP2 fused to microtubule-associated protein 2 (MAP2), prior to vitrification by plunge-freezing. To promote cell growth on EM grids we used gold grids with a slightly thicker carbon film of 49 nm (cf., 17 nm above)^49^ required for cell culture^2^. The change in grid type necessitated a different illumination intensity, and we noticed that the cells act as a cryoprotectant, in agreement with earlier reports^28^; even when surrounding ice displayed devitrification, the ultrastructure inside the cells appeared undamaged. By reducing the laser from 28.5 W/cm^2^ to 20.8 W/cm^2^ we could image 8 out of 23 imaged positions with no visible devitrification, either inside or outside of the cells. Furthermore, 11 out of 23 imaged positions displayed devitrification outside the cells, but no devitrification within cells was detected. Using this new grid type and illumination intensity, we could successfully perform SR-cryoCLEM inside cells with a success rate of ~83% (Fig. 5). We observed activation of rsEGFP2 in the cytoplasm that localised to bundles of microtubules (Fig. 5a–c). When samples were investigated by cryoEM we could successfully trace back bundles of microtubules in thin regions within the cell periphery (Fig. 5d–f). Reconstruction yielded an average localisation precision of 27 nm, in agreement with the values of lipid nanotubes (Supplementary Fig. S5c). We also performed 3D cryo-electron tomography at the same region and used ec-CLEM^50^ to align the tomographic volume with the SR reconstruction (Fig. 5g,h). This allowed us to straightforwardly visualize two microtubule bundles curved around intracellular vesicles, which could be resolved by cryoSMLM but not with diffraction-limited cryoFLM (Fig. 5d,g,h).

## Discussion

In this work, we described the application of SMLM on cryosamples to increase the interpretability of cryoCLEM, and addressed several challenges. Firstly, the increased drift of cryostages had to be mitigated. We have used the commercially available Linkam cryostage and a straightforward image registration method (see Supplementary Note) using fiducial beads to counter instabilities during imaging, and achieved a registration accuracy of ca. 10 nm (Supplementary Fig. S4). Secondly, high intensity laser illumination can cause sample devitrification (Fig. 1), caused by the light absorption of the carbon support film. We have used commercially available thin holey-carbon film grids, C-flats & Quantifoil, and limited laser intensity to 28.5 W/cm^2^ and 20.8 W/cm^2^, respectively. This allowed us to continuously illuminate the C-flat grids for prolonged periods of time without causing any devitrification, and we saw no devitrification in ~83% of regions imaged inside of cells grown on Quantifoil grids. Next, we systematically assayed the behaviour of vitrified (RS)FPs at 77 K. We found that all FPs tested displayed similar photoactivation behaviour (Fig. 2 and Supplementary Figs 1–2). Although we used only 7 FPs (Table 1), we selected these such that different activation mechanisms (positive vs negative switcher, irreversibly-photoactivatable PA-GFP, etc) were investigated. The similar behaviour we observe when vitrified suggests a common switching mechanism of all FPs at 77 K, which may indicate that any FP can be used to perform SR-cryoCLEM. We then demonstrated SR-cryoCLEM on lipid nanotubes labelled with rsEGFP2 and rsFastLime as a proof of concept on isolated, *ex vivo* structures, and achieved a localisation precision of 30 nm (Supplementary Fig. S5). We note that the fluorescence intensity of labelled lipid nanotubes was greater above the holes in the C-flat holey-carbon grids, and the number of photoactivation events was also increased (Supplementary Fig. S7). Whilst this is unlikely due to local heating of the sample, it is currently unknown what causes this effect, although it may be that this could be reduced by using support films other than carbon. To demonstrate the potential of this technique, we next performed SR-cryoCLEM on intact mammalian cells (Fig. 5) and achieved a localisation precision of 27 nm (Supplementary Fig. S5). This was sufficient to allow us to visualize two microtubule bundles split by intracellular vesicles in the periphery of cells (Fig. 5g–h). To our knowledge, this is the first time cryoSR imaging has been performed within intact mammalian cells. Furthermore, the use of vitrification as a fixation method, thereby avoiding artefacts associated with chemical fixation, combined with non-invasive labelling using genetically-encoded (RS)FPs, will allow cryoSMLM to replace conventional room-temperature SR techniques and increase its applicability beyond cryoCLEM. When no successive cryoEM imaging is necessary, sample devitrification might be acceptable, since the structural rearrangements caused by devitrification are likely to be within the resolution range of SR-cryoFLM. Alternatively, samples may be vitrified on non-absorbing support materials not suited for successive cryoEM imaging, such as a solid metal surface with a high heat conductivity, thereby preventing heating and devitrification of cryosamples. With the emerging technique of cryo-focused ion beam milling to image intracellular architectures^51^, SR-cryoCLEM will become an invaluable technique to identify regions of interest with high precision^52^. With the methodology we have described here, SR-cryoCLEM becomes broadly available and applicable, using commercially available equipment and common FPs.

## Materials and Methods

### Expression and purification of fluorescent proteins

Fluorescent proteins Dronpa, rsFastLine, rsEGFP2 and Padron were expressed as described^14,15^. Genes for EGFP^45^, PA-GFP^46^ and mIrisFP^48^ were ordered from Integrated DNA Technologies (Leuven, BE) and cloned into pET45b(+) in frame with an N-terminal His6 tag. Expression in BL21(DE3) cells was followed by purification using Ni-NTA-affinity chromatography. All proteins were buffer-exchanged into 50 mM Tris-HCl (pH 7.5), 150 mM NaCl by repeated concentration and dilution using 30 kDa-cutoff spin concentrators (Amicon, Merck millipore).

### SMLM microscope set-up

We adapted a standard upright widefield fluorescent microscope (Leica DM RXA) for SMLM acquisition. A 405 nm laser (LuxX + 405–120, Omicron) and a 488 nm laser (LuxX + 488–100, Omicron) were used for activation and excitation, respectively, which were coupled in using a polarising-maintaining optical fibre, all from Omicron-Laserage Laserprodukte GmbH, Germany. Standard Leica fluorescent cubes were used (FITC filter or N2.1 filter), with the excitation filters removed from the filter blocks. Fluorescence was collected using either a long-working distance lens (HCX PL Fluotar L 100x/0.75, Leica) in combination with a cryostage (CMS-196, Linkam Scientific, UK), or an oil-immersion lens for experiments at 294 K (PL Fluotar 100x/1.3, Leica). Fluorescent images were recorded by a sCMOS camera (pco.edge 4.2, DVision, Oostakker, Belgium).

Fluorescence emission spectra were recorded by a home-made set-up that was mounted on an extra camera port via a beam-splitter (100-50-0%, Leica). The emitted light was coupled in a multimode optical fibre (M15L01, Thorlabs Inc, USA) by an aspheric glass lens (C340 TMD-A, Thorlabs Inc, USA) and connected to a fibre-optic spectrometer (AvaSpec-2048L, Avantes, the Netherlands). Software written in-house was utilised to read-out the camera, and to synchronise the lasers and camera acquisition.

### Monitoring ice devitrification

For devitrification experiments, freshly glow discharged C-Flat grids (2/1-2C, Electron Microscopy Science, USA) with deionised water were plunge-frozen using a Leica EM-GP, with blotting time 1 sec. Grids were loaded in the cryoFLM set-up, and illuminated with 488 nm laser light as described, for various intensities and durations (Fig. 1). On a single grid up to 10 different conditions could be examined, leaving at least one grid square between locations to avoid influence from previously illuminated areas. It should be noted however, we did not detect any effect on neighbouring grid squares, even after a grid square was illuminated for several minutes using maximum illumination intensity (83.0 W/cm^2^). Furthermore, we note that the intensity threshold is expected to be dependent on experimental setup (e.g. support material and mesh size). Grids were then transferred to a Gatan 626 cryo holder (Gatan, Pleasanton, USA) for inspection with cryoEM. The distinction between devitrified and vitreous samples is immediately apparent in the cryoEM, as is shown in Fig. 1.

### Spectroscopy of FPs at 77 K and 294 K

For spectroscopy experiments at 294 K, glass slides and cover slips were cleaned with water and detergent, rinsed with acetone and dried. Slides were coated with poly-L-lysine (0.1 w/v% in water, Sigma), and 1 μl of 180 μM fluorescent protein in buffer (50 mM Tris-HCl (pH 7.5), 150 mM NaCl) was added on top of the poly-L-lysine layer and covered with a cleaned coverslip. For cryogenic spectroscopy experiments, glow-discharged C-Flat grids (2/1-2 C, Electron Microscopy Science, USA) containing 180 μM of fluorescent protein were plunge-frozen using an EM grid plunger (Leica EM-GP), with blotting time 1 sec.

Samples were imaged on a standard room temperature-stage or a Linkam cryostage (CMS-196, Linkam Scientific, UK), using 405 nm laser light (LuxX − 405 – 120, Omicron-Laserage Laserprodukte GmbH, Germany), and laser dichroic ZT405rdc (Chroma Technology GmbH, Olching Germany) without any additional excitation or emission fluorescent filters.

### Activation and deactivation of FPs

To assess the ability of FPs to be activated at cryogenic conditions, samples were prepared similarly to spectroscopy experiments, with a reduced concentration of 20 μM. For deactivation prior to vitrification, samples were illuminated using a lamp (Excelitas, EXFO X-cite series 120), filtered with a standard GFP excitation filter (Omega Optics, cat. no. 480DF20, bandwith 470–490 nm), for 1 min. Samples were vitrified within 1 minute after deactivation, and either stored in liquid nitrogen or loaded directly in the Linkam cryostage. Then, samples were activated by a 2.5 sec pulse of 405 nm laser light, with intensity 28.5 W/cm^2^. Deactivation was recorded under continuous illumination of 488 nm light (28.5 W/cm^2^) for 20 sec, with a camera exposure time of 50 ms and a 20 ms read-out time after each recorded image. For each FP at least 5 cycles were recorded.

To extract the deactivation rate of the FPs from the first cycle of the recorded activation traces, images were masked to exclude regions from the grid bar and out-of-focus areas. Then, the average intensity of each frame was used, and fitted with either a bi-exponential function^53^ or a mono-exponential function for the traces of EGFP and Padron at 294 K.

### Preparation of lipid nanotubes labelled with FPs

Lipid nanotubes comprised of Galactose-ceramide:Ni-NTA DGS lipids (99:1 mol%) were formed in 50 mM Tris-HCl (pH 7.5), 150 mM NaCl buffer as previously reported^54^ and labelled with His-tagged FPs by incubating the FP and lipid nanotubes at 4 °C for 1 hour.

Labelled lipid nanotubes were applied to the carbon support of freshly glow-discharged C-Flat grids (2/1-2C, Electron Microscopy Science, USA). Grids were then washed three times, to wash away unbound FPs, by blotting from the backside of the grid and quickly rehydrating with fresh buffer. In the last washing step 100 nm tetraspeck beads (ThermoFisher Scientific) were added. The grids were then illuminated with blue light to deactivate the FPs on the grids as described prior to blotting from the reverse side of the grid and plunge-freezing. Grids were stored in liquid nitrogen.

### Preparation of Mammalian Cells Transfected with rsEGFP2-MAP2

Gold quantifoil grids (R 1/4, 200 mesh; Electron Microscopy Science, USA) were glow-discharged and laid in 1 ml cell culture dishes, with the carbon side upwards. 30,000 U2OS cells were seeded in Gibco Dulbecco modified eagle medium (DMEM; ThermoFisher, USA), and incubated at 37 °C in a 5% CO_2_ atmosphere. A plasmid encoding rsEGFP2-MAP2 was generated by exchanging the α-tubulin sequence of pEGFPTub (Clontech) with the MAP2 sequence, amplified from pDONR223-MAP2^55^, before the EGFP sequence was exchanged with rsEGFP2^15^. After 24 hours, cells were transfected with the rsEGFP2-MAP2 plasmid using polyethylenimine (Polysciences, Inc., USA) in Gibco opti-Eagle’s minimum essential medium (ThermoFisher, USA). After 24 hours, the medium was exchanged with fresh DMEM, and cells were fixed by vitrification 24 hours afterwards. Firstly, the cells were washed three times with PBS. Grids were then carefully picked up and 2 µl of 200 nm tetraspeck beads (diluted 1:15 in PBS) added before being placed under the deactivation lamp (as described above) for ca. 1 min. Finally, the cells were plunge-frozen using the Leica EM GP, operated at 37 °C and 95% humidity. Blotting was performed twice for 4 s each from the backside of the grid.

### Cryo-fluorescence microscopy and cryoSMLM

Grids were transferred to the cryostage (CMS-196, Linkam Scientific, UK) and suitable regions on the grid were found by searching whilst illuminating with 488 nm light. When a region was found, its position on the grid was noted for later correlation. For samples that were not pre-deactivated at room temperature, first a deactivation step was performed, by illuminating the sample with 488 nm light for up to 15 min. Next, SR imaging was initiated by first illuminating the sample with an activation pulse (405 nm, 28.5 W/cm^2^), see also Supplementary Fig. S3. Each activation pulse was followed by 2.8 sec of imaging, corresponding to 40 acquisition frames each of 50 ms exposure time with a 20 ms read-out time, under constant exposure to 488 nm light (28.5 W/cm^2^). For the work with cells, laser intensity was reduced to 20.8 W/cm^2^ to account for the thicker carbon layer necessary for cell adherence to grids, and to speed up imaging, 10 acquisition frames per imaging cycle were acquired. For each position, the activation pulse started at 200 ms length, and was increased incrementally to 2 sec, to counter the switching fatigue of the fluorophores. During imaging, regular refocussing was necessary, since axial drift cannot be corrected post acquisition. After cryoFLM acquisition, grids were stored under liquid nitrogen until cryoEM imaging.

### Reconstructing SR images

Fluorescent image stacks from SR data collection were processed to compensate for drift and background (Supplementary Fig. S3). First, images were cropped and aligned to correct for lateral drift, using a home-written registration method based on locating and registration of fluorescent beads, or alternatively on the sample itself (see also Supplementary Fig. S4 and the Supplementary Note). Then, for each activation cycle, the last frame prior to the activation pulse was subtracted from all frames of that cycle. To avoid clipping the background-corrected images, which can introduce artefacts during the single-molecule localisation, a constant value was added to the frames prior to subtracting. Lastly, frames acquired during refocussing were discarded from the data, since they produced erroneous detections (see Supplementary Fig. S8). The resulting stack was then analysed using the software ThunderSTORM^56^. Single molecule events were filtered such that events with high sigma values were ignored for the reconstruction, and with these final locations a SR heatmap was generated, using a SR pixelsize from one sixteenth to one ninth of the original acquired frames. Finally, an overview image of the grid was prepared to facilitate identification of imaged regions in the EM. Localisation precision was calculated by ThunderSTORM, as described in Ovesný *et al*.^56^ and Thompson *et al*.^57^:

$$\langle {({\rm{\Delta }}x)}^{2}\rangle =\frac{2{\sigma }^{2}+{a}^{2}/12}{N}+\frac{8\pi {\sigma }^{4}{b}^{2}}{{a}^{2}{N}^{2}},$$

where $\langle {({\rm{\Delta }}x)}^{2}\rangle$ is the localisation precision in the lateral direction, *σ* the standard deviation of the point spread function of the fitted gaussian function, *a* is the pixel size of the camera, *N* the number of photons for the localised fluorophore, and *b* is the background level.

### Cryo electron microscopy

Grids were transferred to a Gatan 626 cryo holder (Gatan, Pleasanton, USA) and inserted into a Tecnai T12 transmission electron microscope (FEI Company, USA) operating at 120 kV. Images were recorded on a OneView CMOS detector (Gatan Inc., Pleasanton, USA) using low-dose conditions. EM images used for Fig. 5 were collected on a Tecnai F20 transmission electron microscope (FEI Company, USA), operating at 200 kV. Images were recorded on a US 4000 (Gatan Inc, Pleasanton). A tilt series were acquired using the software package Xplore3D (FEI Company, Eindhoven, NL), using a discontinuous tilt scheme from −54 ° to +60 °, using 3 ° increments, under low dose conditions. The tomogram was reconstructed using IMOD and 6 iterations of the simultaneous iterative reconstruction technique.

### Correlation

Precise image correlation was performed using the dedicated software tool ec-CLEM^50^, where fiducial beads are indicated in each imaging modality, after which a precise correlation is calculated by the software. Image interpolation was performed using a nearest neighbour scheme.

## Supplementary information

SUPPLEMENTARY INFO
