# Author Correction: Correlative cryo super-resolution light and electron microscopy on mammalian cells using fluorescent proteins

**DOI:** 10.1038/s41598-022-15037-5

**Published:** 2022-06-28

**Authors:** Maarten W. Tuijtel, Abraham J. Koster, Stefan Jakobs, Frank G. A. Faas, Thomas H. Sharp

**Affiliations:** 1 Section Electron Microscopy, Dept. of Cell and Chemical Biology, Leiden University Medical Center, 2300 RC Leiden, The Netherlands; 2 NeCEN, Gorlaeus Laboratories, Leiden University, 2333 CC Leiden, The Netherlands; 3 Max Planck Institute for Biophysical Chemistry, Dept. of NanoBiophotonics and University Medical Center of Göttingen, Dept. of Neurology, Am Faßberg 11, 37077 Göttingen, Germany

Correction to: *Scientific Reports* 10.1038/s41598-018-37728-8, published online 04 February 2019

The original version of this Article contained errors as a result of a miscalculation in the intensity values required to achieve cryo-super-resolution in vitrified conditions.

In the Introduction,

“We found that cryosamples in our setup could be constantly illuminated for extended periods using a laser intensity of 550 W/cm^2^ without devitrification, although this greatly depends on sample and the support grid used.”

now reads:

“We found that cryosamples in our setup could be constantly illuminated for extended periods using a laser intensity of 28.5 W/cm^2^ without devitrification, although this greatly depends on sample and the support grid used.”

In the Results section, under the subheading ‘Devitrification’,

“We found that devitrification is dominated by the laser intensity, and not the illumination time (Fig. 1c), and when illumination intensity remained at or below 550 W/cm^2^ ca. 95 % of examined areas remained vitreous.”

now reads:

“We found that devitrification is dominated by the laser intensity, and not the illumination time (Fig. 1c), and when illumination intensity remained at or below 28.5 W/cm^2^ ca. 95 % of examined areas remained vitreous.”

In the Results section, under the subheading ‘Cryo super-resolution correlative light and electron microscopy on intact mammalian cells’,

“By reducing the laser from 550 W/cm^2^ to 400 W/cm^2^ we could image 8 out of 23 imaged positions with no visible devitrification, either inside or outside of the cells.”

now reads:

“By reducing the laser from 28.5 W/cm^2^ to 20.8 W/cm^2^ we could image 8 out of 23 imaged positions with no visible devitrification, either inside or outside of the cells.”

In the Discussion section,

“We have used commercially available thin holey-carbon film grids, C-flats & Quantifoil, and limited laser intensity to 550 W/cm^2^ and 400 W/cm^2^, respectively.”

now reads:

“We have used commercially available thin holey-carbon film grids, C-flats & Quantifoil, and limited laser intensity to 28.5 W/cm^2^ and 20.8 W/cm^2^, respectively.”

In the Materials and Methods section, under the subheading ‘Monitoring ice devitrification’,

“It should be noted however, we did not detect any effect on neighbouring grid squares, even after a grid square was illuminated for several minutes using maximum illumination intensity (1600 W/cm^2^).”

now reads:

“It should be noted however, we did not detect any effect on neighbouring grid squares, even after a grid square was illuminated for several minutes using maximum illumination intensity (83.0 W/cm^2^).”

In the Materials and Methods section, under the subheading ‘Activation and deactivation of FPs’,

“Then, samples were activated by a 2.5 sec pulse of 405 nm laser light, with intensity 550 W/cm^2^. Deactivation was recorded under continuous illumination of 488 nm light (550 W/cm^2^) for 20 sec, with a camera exposure time of 50 ms and a 20 ms read-out time after each recorded image.”

now reads:

“Then, samples were activated by a 2.5 sec pulse of 405 nm laser light, with intensity 28.5 W/cm^2^. Deactivation was recorded under continuous illumination of 488 nm light (28.5 W/cm^2^) for 20 sec, with a camera exposure time of 50 ms and a 20 ms read-out time after each recorded image.”

In the Materials and Methods section, under the subheading ‘Cryo-fluorescence microscopy and cryoSMLM’,

“Next, SR imaging was initiated by first illuminating the sample with an activation pulse (405 nm, 550 W/cm^2^), see also Supplementary Fig. S3.”

now reads:

“Next, SR imaging was initiated by first illuminating the sample with an activation pulse (405 nm, 28.5 W/cm^2^), see also Supplementary Fig. S3.”

Under the same subheading,

“Each activation pulse was followed by 2.8 sec of imaging, corresponding to 40 acquisition frames each of 50 ms exposure time with a 20 ms read-out time, under constant exposure to 488 nm light (550 W/cm^2^).”

now reads:

“Each activation pulse was followed by 2.8 sec of imaging, corresponding to 40 acquisition frames each of 50 ms exposure time with a 20 ms read-out time, under constant exposure to 488 nm light (28.5 W/cm^2^).”

Under the same subheading,

“For the work with cells, laser intensity was reduced to 400 W/cm^2^ to account for the thicker carbon layer necessary for cell adherence to grids, and to speed up imaging, 10 acquisition frames per imaging cycle were acquired.”

now reads:

“For the work with cells, laser intensity was reduced to 20.8 W/cm^2^ to account for the thicker carbon layer necessary for cell adherence to grids, and to speed up imaging, 10 acquisition frames per imaging cycle were acquired.”

The original version of this Article also contained an error in the legend of Figure 1.

“Damage of vitreous water caused by 488 nm laser illumination. The specimen shown is a thin holey-carbon film with regularly spaced circular openings supporting a thin film of vitrified water. (**a**) After 30 minutes of constant illumination using an intensity of 550 W/cm^2^, the ice remains vitreous. (**b**) When increasing the intensity to 650 W/cm^2^, after 5 min constant illumination a clear region of devitrified ice is observed. (**c**) Assessing laser-induced damage of vitreous water after constant illumination using various intensities and durations. For each condition, three replicates are shown. (**d**,**e**) Low (**d**) and high (**e**) magnification cryoEM images of vitreous water illuminated for 60 sec at the following intensities: i 600 W/cm^2^; ii 800 W/cm^2^; iii 1000 W/cm^2^; iv 1200 W/cm^2^; v 1600 W/cm^2^. (**f**) Schematic representation of vitreous (green), devitrified (red) or sublimated/dry (white) areas, corresponding with panels d and e. Scalebars: 1 µm in a,b,e and 20 µm in d.”

now reads:

“Damage of vitreous water caused by 488 nm laser illumination. The specimen shown is a thin holey-carbon film with regularly spaced circular openings supporting a thin film of vitrified water. (**a**) After 30 minutes of constant illumination using an intensity of 28.5 W/cm^2^, the ice remains vitreous. (**b**) When increasing the intensity to 33.7 W/cm^2^, after 5 min constant illumination a clear region of devitrified ice is observed. (**c**) Assessing laser-induced damage of vitreous water after constant illumination using various intensities and durations. For each condition, three replicates are shown. (**d**,**e**) Low (**d**) and high (**e**) magnification cryoEM images of vitreous water illuminated for 60 sec at the following intensities: i, 31.1 W/cm^2^; ii, 41.5 W/cm^2^; iii, 51.9 W/cm^2^; iv, 62.2 W/cm^2^; v, 83.0 W/cm^2^. (**f**) Schematic representation of vitreous (green), devitrified (red) or sublimated/dry (white) areas, corresponding with panels d and e. Scalebars: 1 µm in a,b,e and 20 µm in d.”

The original Article has been corrected.

