# Supplementary material for: Correlative cryo super-resolution light and electron microscopy on mammalian cells using fluorescent proteins
Source: Sci Rep. 2019 Feb 4;9:1369. doi: 10.1038/s41598-018-37728-8 (PMC6362030; doi:10.1038/s41598-018-37728-8)
Supplement: Supplementary file 1 — SUPPLEMENTARY INFO [file 41598_2018_37728_MOESM1_ESM.pdf]

SUPPLEMENTARY INFORMATION

Correlative cryo super-resolution light and electron microscopy on mammalian cells using fluorescent proteins

Maarten W. Tuijtel[1], Abraham J. Koster[1,2], Stefan Jakobs[3], Frank G. A. Faas[1,*], Thomas H. Sharp[1,*]

[1]Section Electron Microscopy, Department of Cell and Chemical Biology, Leiden University Medical Center, 2300 RC Leiden, The Netherlands

[2]NeCEN, Gorlaeus Laboratories, Leiden University, 2333 CC Leiden, The Netherlands

[3]Department of NanoBiophotonics, Max Planck Institute for Biophysical Chemistry, Am Faßberg 11, 37077 Göttingen, Germany

*To whom correspondence should be addressed: F.G.A.Faas@lumc.nl, T.Sharp@lumc.nl

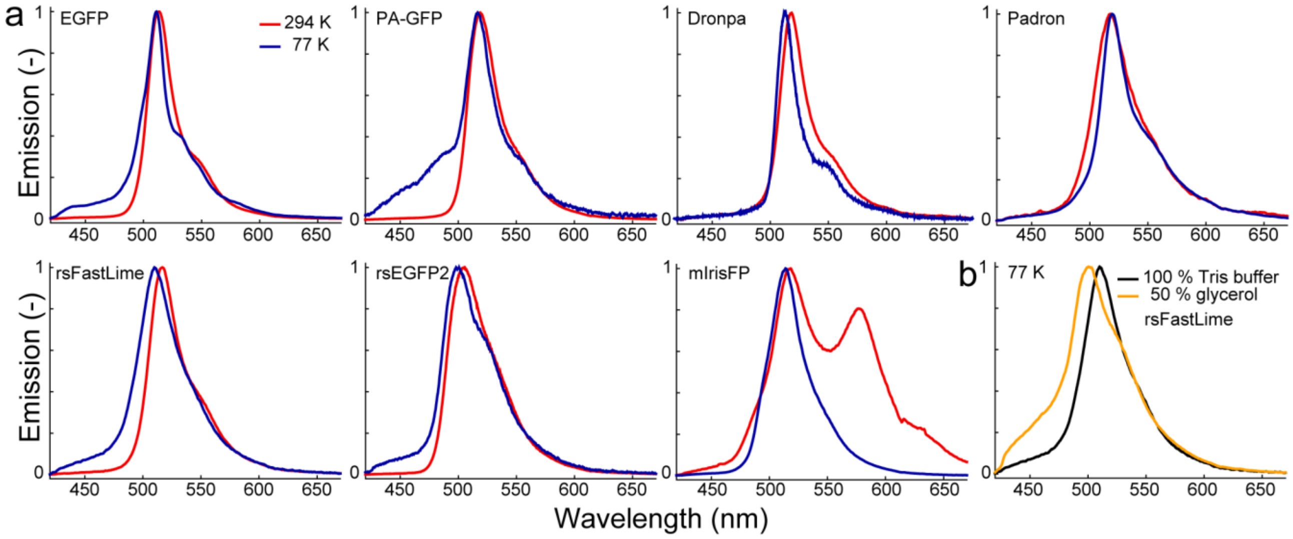

Supplementary Fig. S1. Emission spectra of FPs at both 77K and 294 K. **a**, Emission spectra of FPs in aqueous Tris buffer at 294 K (red lines) and vitrified in Tris buffer at 77 K (blue lines). **b**, Emission spectra of vitrified rsFastLime at 77 K, in Tris buffer (black line) and with 50% of glycerol added as cryoprotectant (orange line).

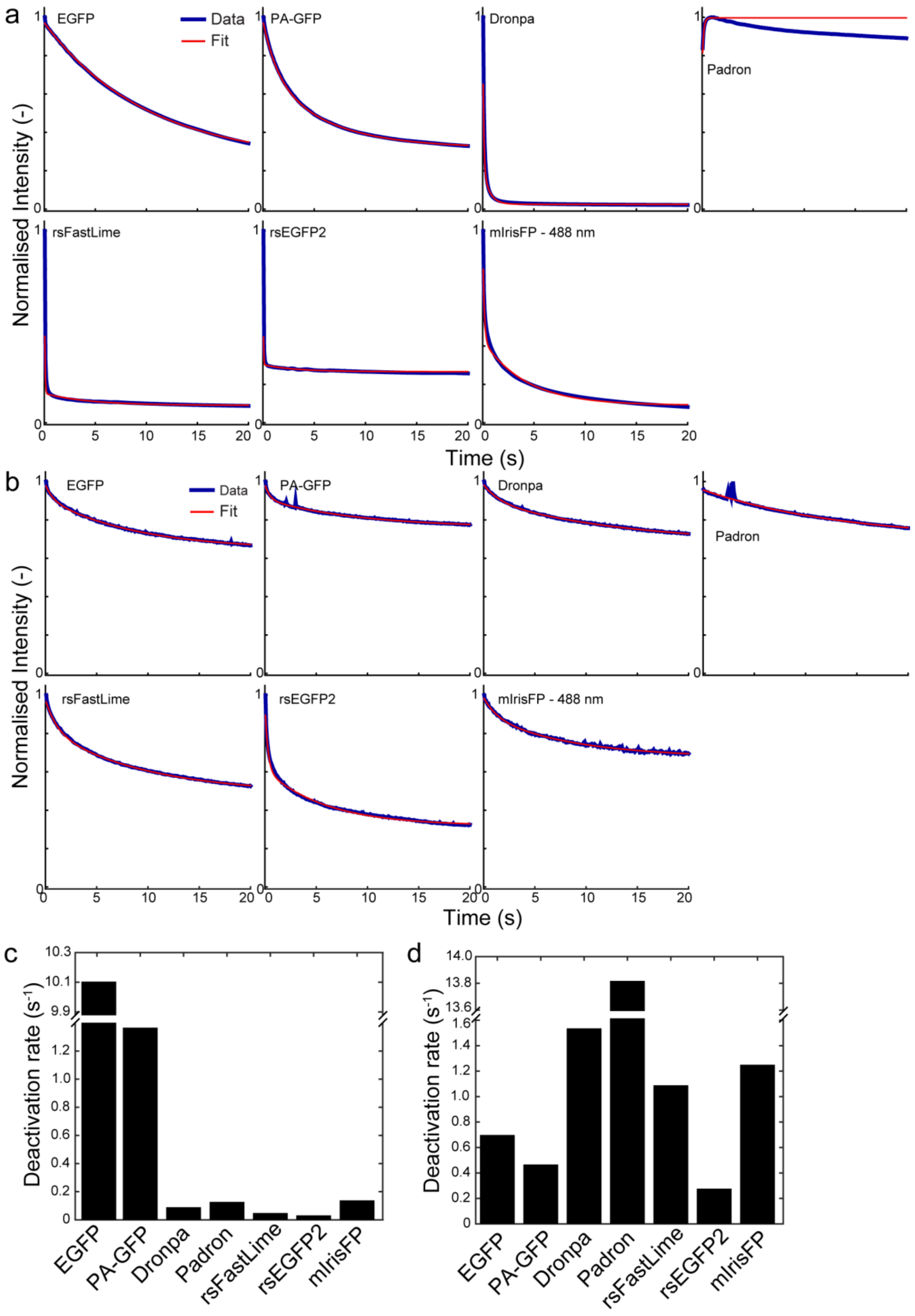

Supplementary Fig. S2. Determining the deactivation rate of FPs. **a**, Deactivation traces (blue) and bi-exponential fits (red) of the deactivation of FPs at 294 K, after a single photoactivation pulse. **b**, Deactivation traces (blue) and (bi-)exponential fits (red) of the deactivation of vitrified FPs at 77 K, after a single photoactivation pulse. **c,d,** The fast phase of the (bi-)exponential fit for FPs at 294 K (**c**) and vitrified FPs at 77 K (**d**) from the traces shown in panels **a,b**.

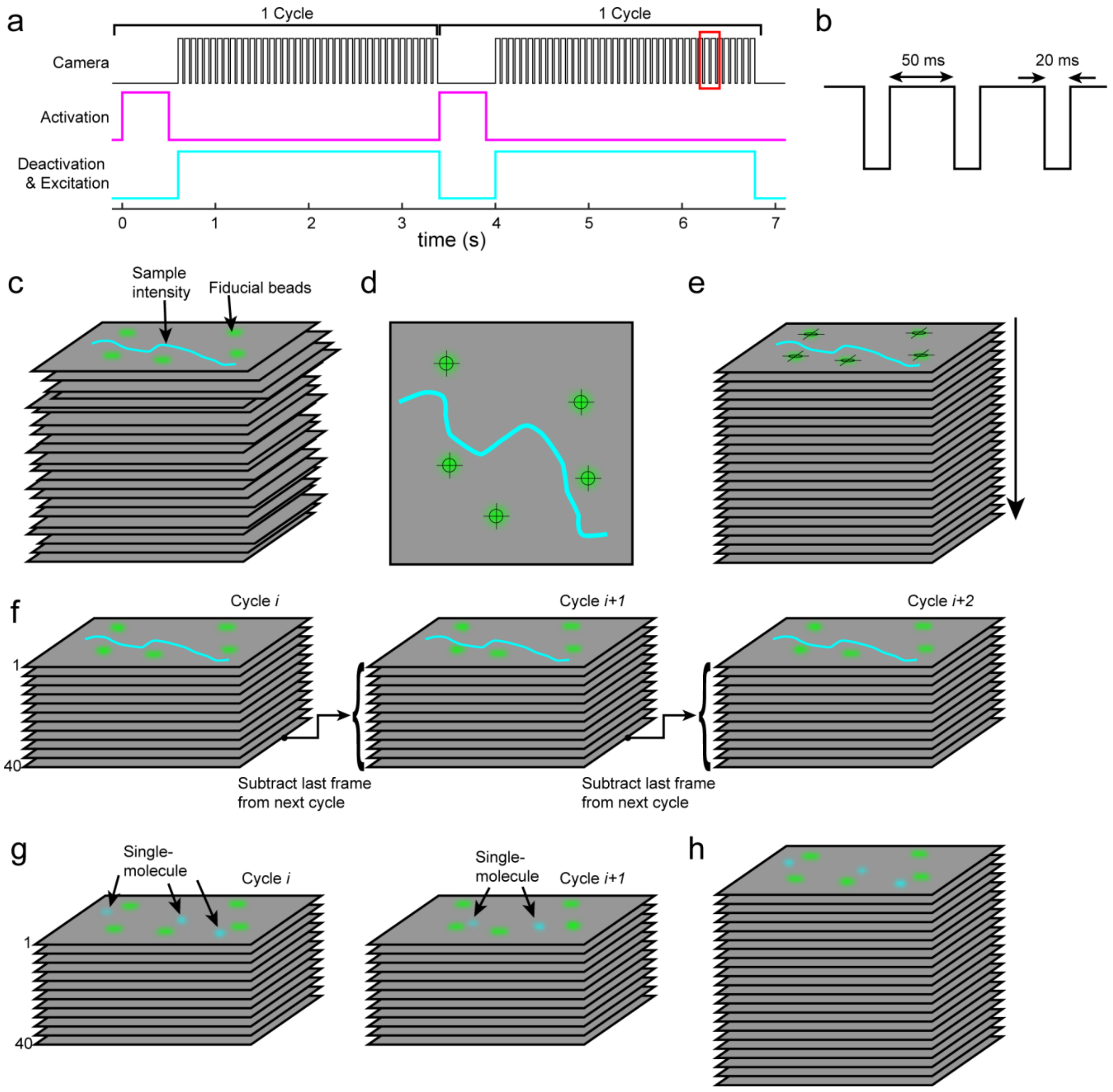

Supplementary Fig. S3. Overview of the cryoSMLM acquisition parameters and data analysis workflow. **a**, First, a small subset of FPs is activated using a pulse from the 405 nm activation laser. A 200 ms pulse is used at the start of acquisition, rising to 2 sec, which is increased during acquisition to counter switching fatigue. Next, the sample is illuminated with the 488 nm excitation and deactivation laser, and fluorescence is recorded using 40 frames of 50 ms exposure time. **b**, Zoom of the region shown in red in panel **a**. An exposure time of 50 ms is followed by 20 ms dead-time in which the camera data is transferred to and stored on the computer. **c**, Schematic representation of the unaligned data-stack after cropping. Fiducial beads (represented in green) are present on the sample for drift correction and correlation. Alternatively, the sample intensity itself (shown in cyan) can be used. **d**, Fiducial beads are detected in all images and **e**, Aligned using a rigid transformation, see also **Supplementary Fig. S4**. **f**, The last frame of each cycle is subtracted from all frames of the subsequent cycle to only show newly activated FPs as is depicted in **g**. **h**, Finally, an aligned stack of single-molecule data can be processed using ThunderSTORM[1].

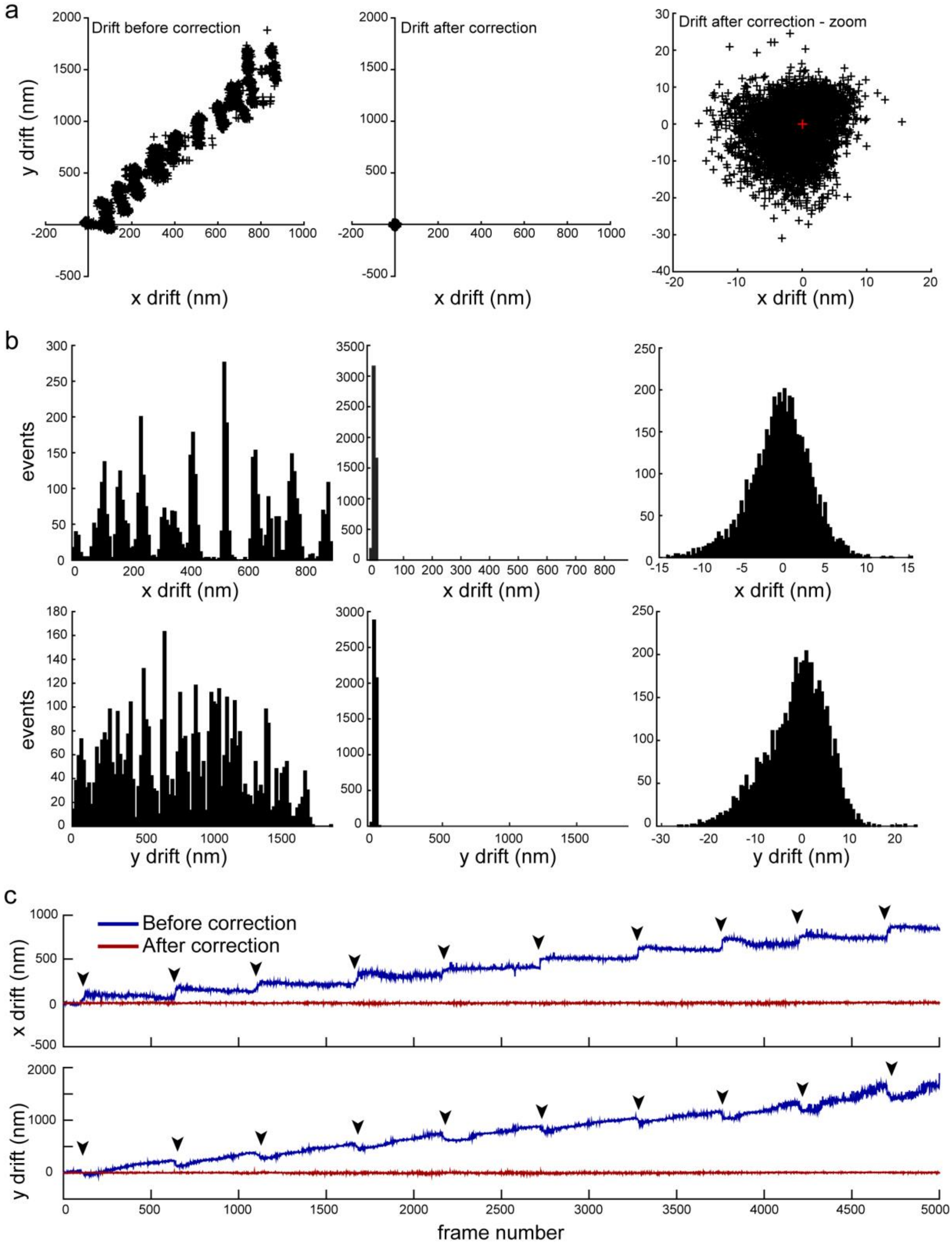

Supplementary Fig. S4. Measuring the drift correction, using fiducial beads. Note that the measurement and correction of drift were performed independently. **a**, Scatter plots of the drift in x (horizontal axis) and y (vertical axis) before (left) and after (centre) correction, and a zoomed image of the after correction (right). **b**, Histogram representation of the same data shown in panel **a**, for x-coordinate (top) and y-coordinate (bottom) for before (left) and after (centre) correction, and a zoomed image of the data after correction (right). **c**, The drift in x-coordinate (top) and y-coordinate (bottom) versus acquisition frame, before (blue line) and after (red line) drift correction, of the same data shown in **a**,**b**. Black arrowheads point to refilling of the imaging chamber of the cryostage with liquid nitrogen.

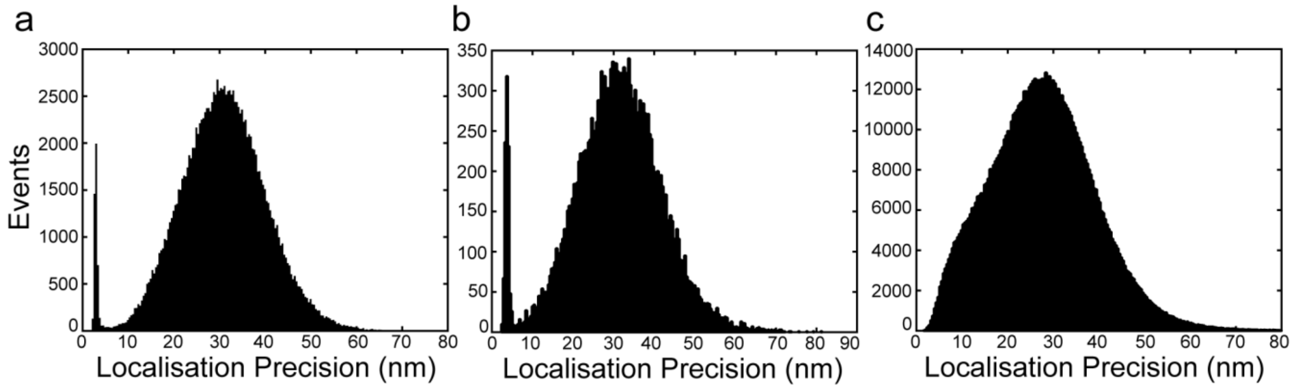

Supplementary Fig. S5. Localisation precision. **a**, Histogram of the localisation precision of the data shown in **Fig. 4a**, where rsEGFP2 was used labelled to lipid nanotubes. **b**, Histogram of the localisation precision of the data shown in **Supplementary Fig. S6**, where rsFastLime was used labelled to lipid nanotubes. **c**, Histogram of the localisation precision of the data shown in **Fig. 5**, where rsEGFP2 was used as fluorophore expressed inside mammalian cells.

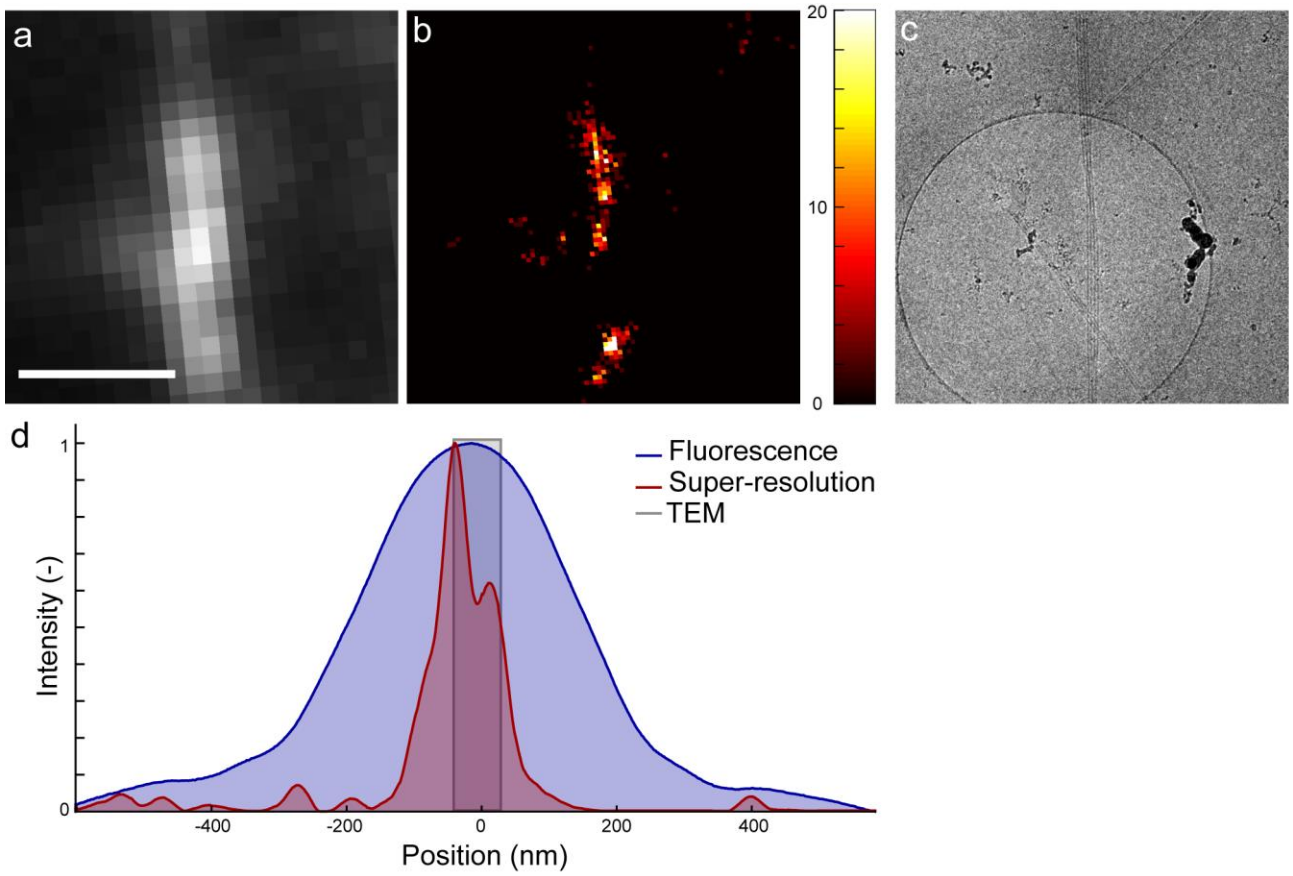

Supplementary Fig. S6. Cryo super-resolution using rsFastLime-labelled lipid nanotubes. **a**, Diffraction-limited cryoFLM image, **b**, CryoSMLM reconstruction, where the colourmap represents the number of localised single-molecules per SR pixel, and **c**, CryoEM image of a bundle of three lipid nanotubes labelled with rsFastLime. **d**, Averaged intensity profile drawn across the bundle of lipid nanotubes shows a clear increase in resolution.

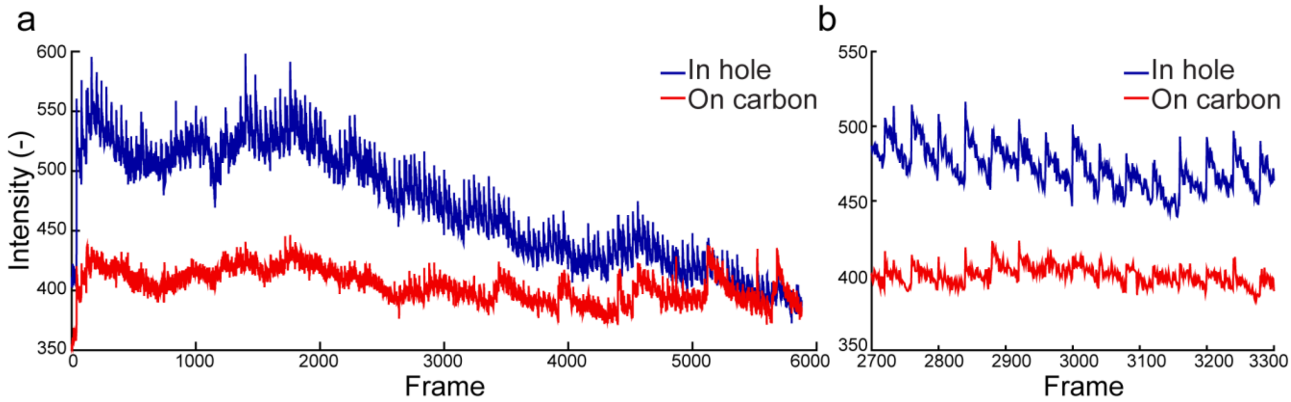

Supplementary Fig. S7. Fluorescent intensity during photoactivation. **a**, Fluorescent intensity of a region on the carbon support film (red line) and inside a hole in the support film (blue line), when acquiring cryoSMLM data. **b**, Zoom of the data shown in **a**. Both fluorescent intensity and photoactivation are stronger inside a hole then on the carbon layer.

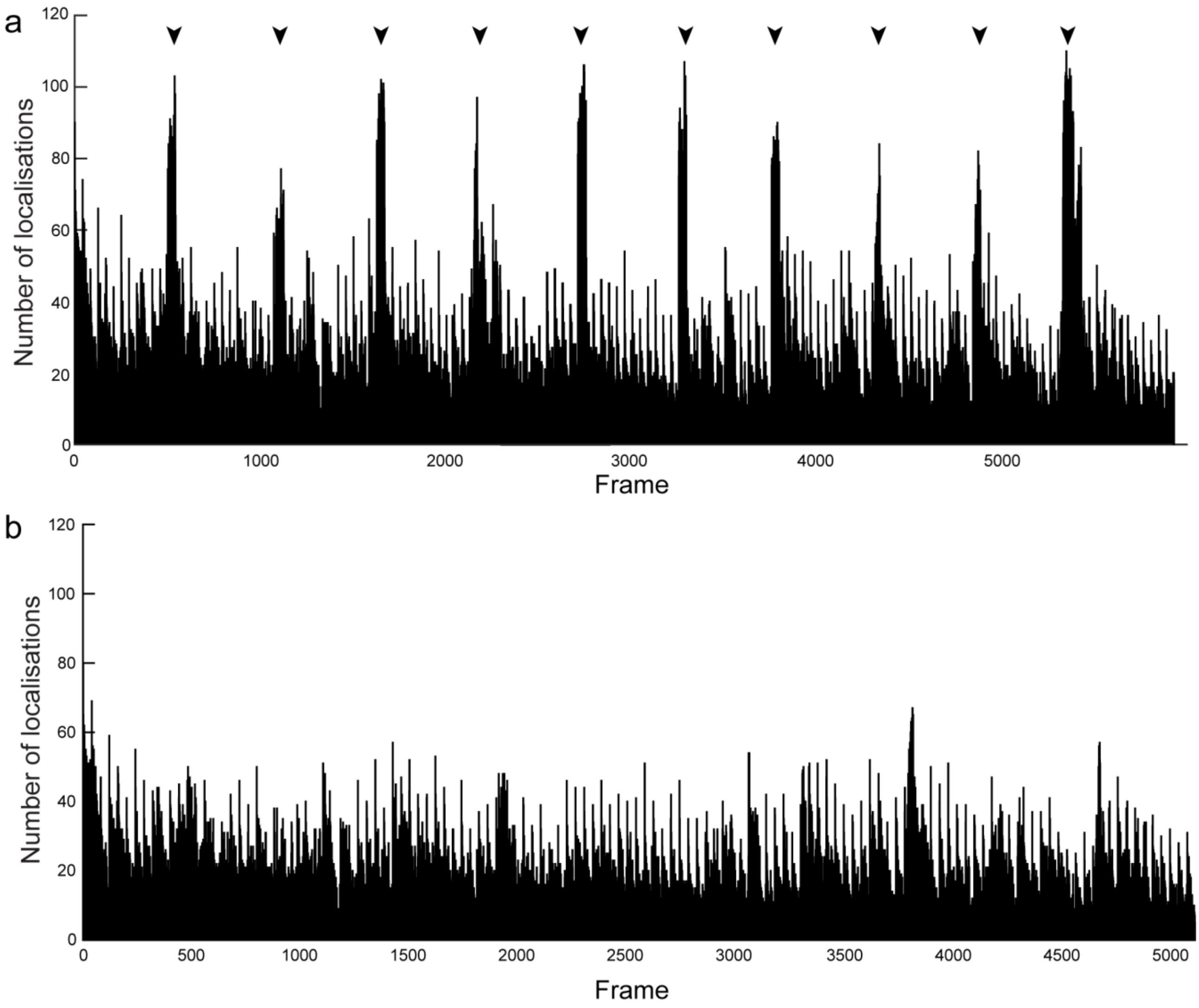

Supplementary Fig. S8. Number of localisations versus frame number. **a**, Refocussing during acquisition can clearly be seen as erroneous single-molecule detections (black arrowheads). **b**, Number of localisations per frame after out-of-focus frames are discarded.

Supplementary Table S1. Single molecule statistics and data on resolution representation. For each dataset, the average number of photons (*N*), localisation precision (σ), Nyquist resolution based on labelling density (Nyq), overall structural resolution (*R*), and the Fourier ring correlation (FRC)[2]. When determining the resolution of a SR image, several factors are significant. Firstly, the localisation precision of detected single-molecule events (σ). Secondly, to fully resolve a structure, the Nyquist sampling theory requires at least two data points per smallest structural feature[3], so the Nyquist-resolution (Nyq) is determined by the labelling density (or more precisely the detection density): Nyq = 2 / $\sqrt{\rho}$, with ρ the sample density in fluorophores/nm^2 [4,5]. To assess the structural resolution (also known as spatial resolution), of the cryoSMLM images, we determined the labelling density, as previously described[4], where from all localised molecules the distance to its 20 closest neighbouring localisations were calculated. We took care to exclude localisations in the same imaging cycle, since they are likely to be the same activated molecule. The overall structural resolution (*R*) was then calculated as $R = \sqrt{(\sigma^2 + \text{Nyq}^2)}$.

Another method to determine the resolution of a SR image is the Fourier ring correlation (FRC)[2,6], which we also calculated using a matlab script provided by Nieuwenhuizen *et al.*[2]. FRC values are always larger than the structural resolution as calculated above[2]. We specifically note that when performing SR-cryoCLEM, the localisation precision is the most important parameter, since the cryoEM provides ultrahigh structural resolution. The main purpose of performing cryoSMLM is to locate regions of interest with high resolution and precision, to be further and with more detail investigated by cryoEM.

**Dataset**	**FP**	***N* (photons)**	**σ (nm)**	**Nyq (nm)**	***R* (nm)**	**FRC (nm)**
**Figure 4a-d**	rsEGFP2	1481	30	60	67	190
**Figure 4e-h**	rsEGFP2	1054	31	70	76	259
**Figure 4i-l**	rsEGFP2	1436	30	39	49	166
**Supplementary Figure 4**	rsFastLime	940	31	104	109	123

Supplementary Note For image registration to correct for the lateral drift of the cryostage, two two methods were used. Firstly, the drift was corrected using the fluorescent intensity of the sample, using the following code sequence in Matlab (Mathworks), with the dipimage plugin[7] installed. The original unaligned data stack is named "`Stack`" and the shift of the images is stored in "`Shifts`".

```
Shifts(1,:)=[0,0]
slice1=squeeze(Stack (:,:,0));
for ii=1:(size(Stack,3)-1)
   slice=squeeze(Stack(:,:,ii));
   Shifts(ii+1,:)=findshift(slice1,slice,'iter');
end
```

Alternatively, the shift was calculated by using fiducial beads that were visible throughout the data stack. These beads were isolated, their precise position for each frame determined by ThunderSTORM, and therefrom the image shifts ("`Shifts`") was calculated, taking into account the change from coordinates (ThunderSTORM) to pixels (Matlab), using the pixelsize of the camera.

For both methods, the following code sequence was then run in Matlab (Mathworks) to align the unaligned data ("`Stack`"), using "`Shifts`" to produce the aligned data stack "`Stack_align`".

```
Stack_align=Stack;
for ii =1:(size(Stack,3)-1)
   Stack_ align(:,:,ii)=shift(squeeze(Stack(:,:,ii)),[Shifts(ii+1,1),Shifts(ii+1,2)]);
end
```

Supplementary References

1 Ovesný, M., Křížek, P., Borkovec, J., Švindrych, Z. & Hagen, G. M. ThunderSTORM: a comprehensive ImageJ plug-in for PALM and STORM data analysis and super-resolution imaging. *Bioinformatics* **30**, 2389-2390 (2014).

2 Nieuwenhuizen, R. P. *et al.* Measuring image resolution in optical nanoscopy. *Nature methods* **10**, 557-562 (2013).

3 Shannon, C. E. Communication in the presence of noise. *Proceedings of the IRE* **37**, 10-21 (1949).

4 Kaufmann, R. *et al.* Super-resolution microscopy using standard fluorescent proteins in intact cells under cryo-conditions. *Nano Letters* **14**, 4171-4175 (2014).

5 Kaufmann, R. *et al.* Visualization and quantitative analysis of reconstituted tight junctions using localization microscopy. *PloS one* **7**, e31128 (2012).

6 Banterle, N., Bui, K. H., Lemke, E. A. & Beck, M. Fourier ring correlation as a resolution criterion for super-resolution microscopy. *Journal of structural biology* **183**, 363-367 (2013).

7 Hendriks, C. L., Van Vliet, L., Rieger, B., van Kempen, G. & van Ginkel, M. DIPimage: a scientific image processing toolbox for MATLAB. *Quantitative Imaging Group, Faculty of Applied Sciences, Delft University of Technology, Delft, The Netherlands* (1999).
